# PHD2 deletion in endothelial or arterial smooth muscle cells reveals vascular cell type-specific responses in pulmonary hypertension and fibrosis

**DOI:** 10.1007/s10456-021-09828-z

**Published:** 2022-01-08

**Authors:** Harri Elamaa, Mika Kaakinen, Marjut Nätynki, Zoltan Szabo, Veli-Pekka Ronkainen, Ville Äijälä, Joni M. Mäki, Risto Kerkelä, Johanna Myllyharju, Lauri Eklund

**Affiliations:** 1 Oulu Centre for Cell-Matrix Research, Faculty of Biochemistry and Molecular Medicine, University of Oulu, Oulu, Finland; 2 Biocenter Oulu, University of Oulu, Oulu, Finland; 3 Medical Research Center Oulu, Research Unit of Biomedicine, University of Oulu and University Hospital Oulu, Oulu, Finland

**Keywords:** Pulmonary hypertension, Fibrosis, Basement membrane, Endothelial cell, Smooth muscle cell, Extracellular matrix, Hypoxia

## Abstract

Hypoxia plays an important regulatory role in the vasculature to adjust blood flow to meet metabolic requirements. At the level of gene transcription, the responses are mediated by hypoxia-inducible factor (HIF) the stability of which is controlled by the HIF prolyl 4-hydroxylase-2 (PHD2). In the lungs hypoxia results in vasoconstriction, however, the pathophysiological relevance of PHD2 in the major arterial cell types; endothelial cells (ECs) and arterial smooth muscle cells (aSMCs) in the adult vasculature is incompletely characterized. Here, we investigated PHD2-dependent vascular homeostasis utilizing inducible deletions of PHD2 either in ECs (*Phd2*^∆ECi^) or in aSMCs (*Phd2*^∆aSMC^). Cardiovascular function and lung pathologies were studied using echocardiography, Doppler ultrasonography, intraventricular pressure measurement, histological, ultrastructural, and transcriptional methods. Cell intrinsic responses were investigated in hypoxia and in conditions mimicking hypertension-induced hemodynamic stress. *Phd2*^∆ECi^ resulted in progressive pulmonary disease characterized by a thickened respiratory basement membrane (BM), alveolar fibrosis, increased pulmonary artery pressure, and adaptive hypertrophy of the right ventricle (RV). A low oxygen environment resulted in alterations in cultured ECs similar to those in *Phd2*^∆ECi^ mice, involving BM components and vascular tone regulators favoring the contraction of SMCs. In contrast, *Phd2*^∆aSMC^ resulted in elevated RV pressure without alterations in vascular tone regulators. Mechanistically, PHD2 inhibition in aSMCs involved  actin polymerization -related tension development via activated cofilin. The results also indicated that hemodynamic stress, rather than PHD2-dependent hypoxia response alone, potentiates structural remodeling of the extracellular matrix in the pulmonary microvasculature and respiratory failure.

**Supplementary Information:**

The online version contains supplementary material available at 10.1007/s10456-021-09828-z.

## Introduction

In the vasculature oxygen-sensing systems provide an important mechanism to induce angiogenic growth and to adjust blood flow via arterial vasodilatation and constriction. The mechanism involves the transcription factor HIF [1], the stability of which is mainly controlled by prolyl 4-hydroxylase-2 also named as EGNL1 or PHD2. The importance of PHD2-dependent intrinsic responses in two major arterial cell types, endothelial (ECs) and arterial smooth muscle cells (aSMCs), in chronic vascular pathologies in adults are incompletely characterized.

A loss-of-function *Phd2* allele in the germline resulted in embryonic lethality between embryonic (E) days E12.5 and E14.5 due to dysmorphogenesis of the placental labyrinth layer that was associated with poorly vascularized placenta and incomplete cardiac development [2]. As the cardiac phenotype could possibly develop secondary to the placental defect the exact role of PHD2 in vascular development is not completely understood in mice. Nevertheless, in *Xenopus leavis* embryo development, which is not placenta dependent, suppression of *Phd2* led to decreased formation of embryonic blood vessels [3]. Interestingly, in postnatal mice inducible global deletion of the floxed *Phd2* allele using the ubiquitously expressed *Rosa26*^CreERT2^ deleter mouse line at six weeks of age resulted in excessive angiogenesis and dilation of blood vessels [4], severe polycythemia causing venous congestion, dilated cardiomyopathy and increased mortality [5]. Collectively these previous studies indicated that PHD2 activity is needed for both developmental angiogenesis and in vascular homeostasis after birth and that PHD2 has differential roles in embryonic and in adult cardiovascular system.

The importance of PHD2 in ECs has been addressed by crossing the floxed *Phd2* allele with *Tie2-*Cre [6] and *VE-Cadherin-*Cre transgenes [6–8]. *Phd2* deletion by these Cre drivers resulted in obliterative arterial remodeling in the lungs [6–8] and kidneys [7] as well as pulmonary arterial hypertension (PAH) and renal fibrosis when analyzed in adulthood. In *Tie2* and *VE-Cadherin* promoter driven mice Cre is expressed form early development starting at E7.5 in blood and lymphatic ECs [9, 10]. The promoters are not exclusively expressed in ECs, but also in hematopoietic cells, some circulating cells, fibroblasts and in the mesenchymal cells of the atrioventricular canal and in part of the proximal cardiac outflow tract [9–11]. As cardiac perivascular cells can develop from ECs after E7.5 [12] *Tie2-*Cre and *VE-Cadherin-*Cre can also result in *Phd2* deletion in a wider manner including pericytes and in vascular SMCs. Understanding the importance of PHD2 arterial smooth muscle cells (aSMCs), which are the key cell type in the regulation of pulmonary arterial pressure, is of particular interest due to link between PHD2 deficiency and PAH. In a previous study PHD2 [13] was deleted using inducible generic SMC deleter *Smmhc*-CreER^T2^ mouse line [14] that enhanced pulmonary and cardiovascular pathologies in hypoxia and in hypertensive mice, but not in normoxia. From a translational point of view, deletion of PHD2 in aSMCs would be especially informative to better understand the pathophysiology of PAH, however, the current limitation is that there is no selective Cre driver line for conditional gene modifications in aSMCs [15]. In addition, due to differential effects of PHD2 deletion in embryonic angiogenesis and adult vasculature, the current models are inadequate to investigate the importance of endothelial PHD2 in vascular homeostasis in quiescent endothelium. To overcome these limitations, we generated *Phd2*^∆ECi^ mice to address the importance of PHD2 in ECs after embryonic development. In addition, to investigate PHD2 deficiency in pulmonary aSMCs, we generated *Phd2*^∆aSMC^ mice using a novel deleter mouse line (*Angpt4*^Cre^) that in the lungs drives Cre expression in aSMCs starting in postnatal mice.

The PHD/HIF signaling pathway is involved in a large number of developmental processes, in adult homeostasis and pathologies having either protective or deleterious effects [16–19], and it has also emerged as an important target for drug development [20]. Clearly, in depth investigation of the cell type-specific roles of PHD2 in complex systems is biologically interesting and clinically highly relevant. In our study, longitudinal investigation of *Phd2*^∆ECi^ revealed progressive lung disease characterized by increased pulmonary artery pressure, interstitial fibrosis, and compensatory RV hypertrophy. Surprisingly, and in contrast to previous models where PHD2 was deleted from ECs starting from embryogenesis, *Phd2*^∆ECi^ resulted in an increased pulmonary artery pressure gradient and cardiac hypertrophy without structural remodeling of the pulmonary arteries (i.e. no alterations in aSMC proliferation, arterial wall or periarterial extracellular matrix). Our study is also the first to show that long-term PHD2 deletion in ECs results in alveolar capillary basement membrane (BM) thickening and increased basement membrane (BM) collagen IV expression that may impair gas exchange and thus aggravate symptoms in PAH. Analysis of *Phd2*^∆aSMC^ mice, for the first time, revealed the PHD2-dependent hypoxia response in pulmonary arterial SMCs in vivo indicating novel, autonomous role for PHD2 in aSMCs in maintaining vascular tone. Mechanistically, PHD2 inhibition in aSMCs was associated with cofilin dephosphorylation which has been shown to enhance actin polymerization and related tension development [21]. Finally, we dissected the contribution of PHD2-dependent HIF signaling from the mechanical stimulus emerging from elevated blood pressure.

## Results

### Generation of inducible postnatal deletion of *Phd2* in endothelial cells (*Phd2*^∆ECi^)

**Fig. 1:**
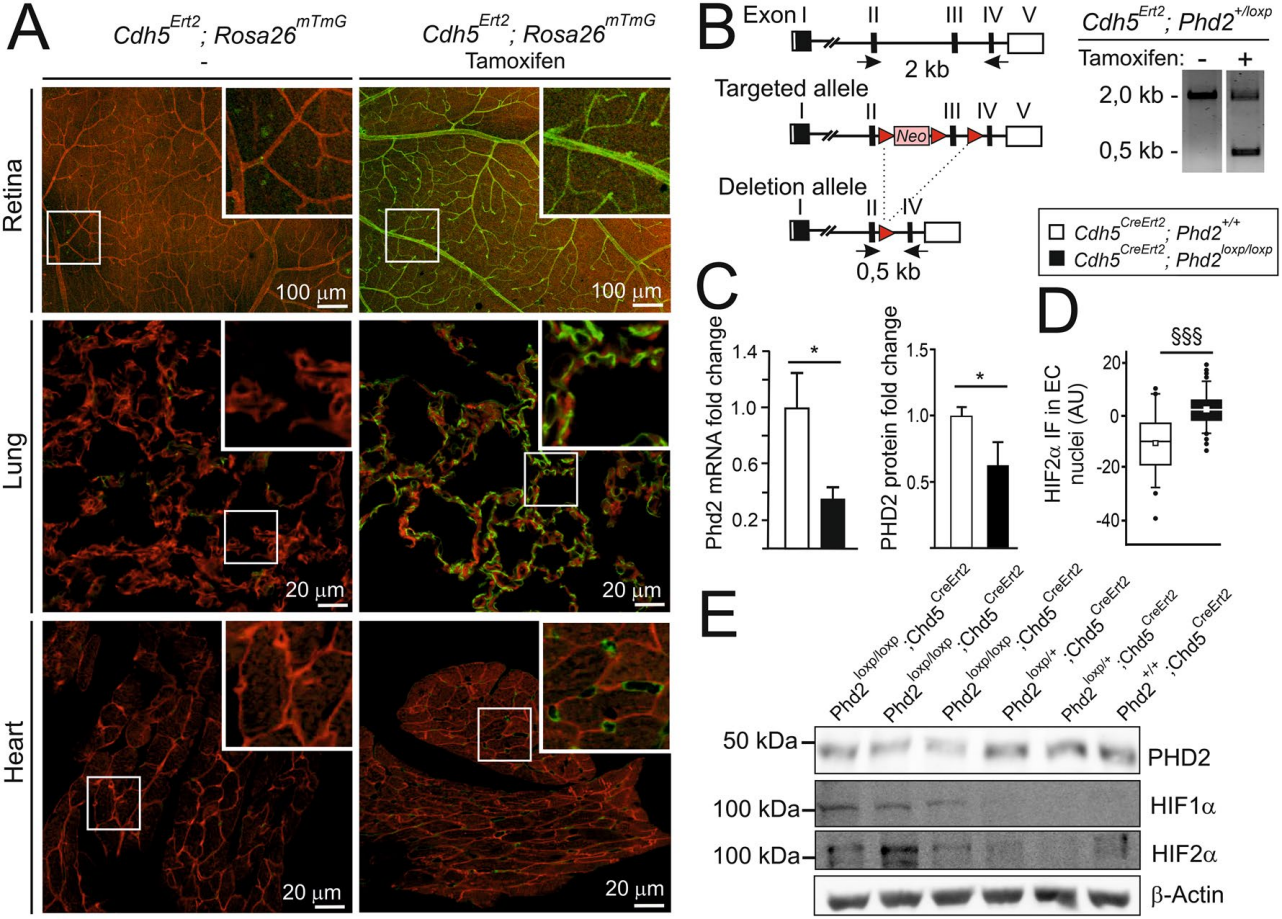
Endothelial cell-specific Cre/loxP recombination and PHD2 deletion in *Phd2*^ΔECi^ mice. **A** The tissue samples collected from 10-month-old mice and imaged with the confocal microscope. In *Cdh5*^CreErt2^; *Rosa26*^mT/mG^ mice, mTomato (red) is ubiquitously expressed until the tamoxifen-induced Cre/loxP recombination that induces GFP (green) expression. Tamoxifen-treated mice (right) show  GFP-signal in ECs, whereas in the untreated control (left) only weak and non-EC background is detected. **B** WT (up), targeted *Phd2* locus (middle) and Cre-loxP mediated deletion of exon 3 encoding the catalytic domain of PHD2 (bottom). Red triangles, *loxP* sites; arrows, location of PCR primers to detect WT and deletion alleles. In tamoxifen-treated *Cdh5*^CreErt2^*; Phd2*^+/loxP^ mice genomic PCR (right) indicates *Phd2* deletion (0.5 kb PCR product). **C** mRNA and protein levels quantified by qPCR and Western blot (**E**). **D** Relative HIF2α immunofluorescence intensity in arterial EC nuclei, *n* = 56 and 122 nuclei from two control and four tamoxifen-treated *Cdh5*^CreErt2^*; Phd2*^loxP/loxP^ mice. Median (line), average (square), 75th quartile (box), 5th and 95th percentile (whiskers), outliers (•). **E** Western blot analysis of 3-month-old mouse lung lysates showed diminished PHD2 protein level and stabilization of HIF1α and HIF2α in tamoxifen-treated *Cdh5*^CreErt2^*; Phd2*^loxP/loxP^ mice. Note that both mRNA and protein analysis (**C**, **E**) detect residual non-endothelial PHD2 as expected. Mean ± SD. *P < 0.05 in *t*-test, ^§§§^*P* < 0.001 in Mann–Whitney test

To generate an inducible deletion of *Phd2* in ECs, a floxed *Phd2* allele was crossed with a *Chd5*^CreERT2^ driver line [22], which generates complete recombination of the floxed allele in various blood vascular beds including retina, liver, heart, lung, kidney and brain. Tamoxifen was administered as detailed in the Material and Methods starting at one month of age and the mice were studied at 3, 6, and 10 months of age. The efficacy of EC-specific recombination between *loxP* sites was confirmed in the *Chd5*^CreERT2^; *Rosa26*^mT/mG^ reporter line as described previously [23]. Based on analysis of the retinal flat mounts, lung and heart tissue sections, Cre/loxP recombination was complete in *Chd5*^CreERT2^; *Rosa26*^mT/mG^ ECs and did not occur in other cell types (Fig. 1A). Based on PCR analysis of the genomic sequence over the floxed region (Fig. 1B), deletion of the *Phd2*^flox^ allele occurred in tamoxifen-treated *Chd5*^CreERT2^; *Phd2*^flox^ mice resulting in a reduction in PHD2 expression in the lungs as expected (Fig. 1C). Keeping in mind that about 30% of all lung cells are ECs [24], a 26.1% reduction in PHD2 protein in the total lung preparation from tamoxifen-treated *Chd5*^CreERT2^; *Phd2*^flox/flox^ mice (*Phd2*^∆ECi^ onwards) indicated almost complete deletion of *Phd2* in ECs resulting in stabilization of HIFs (Fig. 1D and E, Supplemental Figs. 1, 2). Comparison of *Phd2*^∆ECi^ with previously reported non-inducible transgenic *Chd5-Cre* mice [8, 25] indicated similar deletion efficacies.

**Fig. 2:**
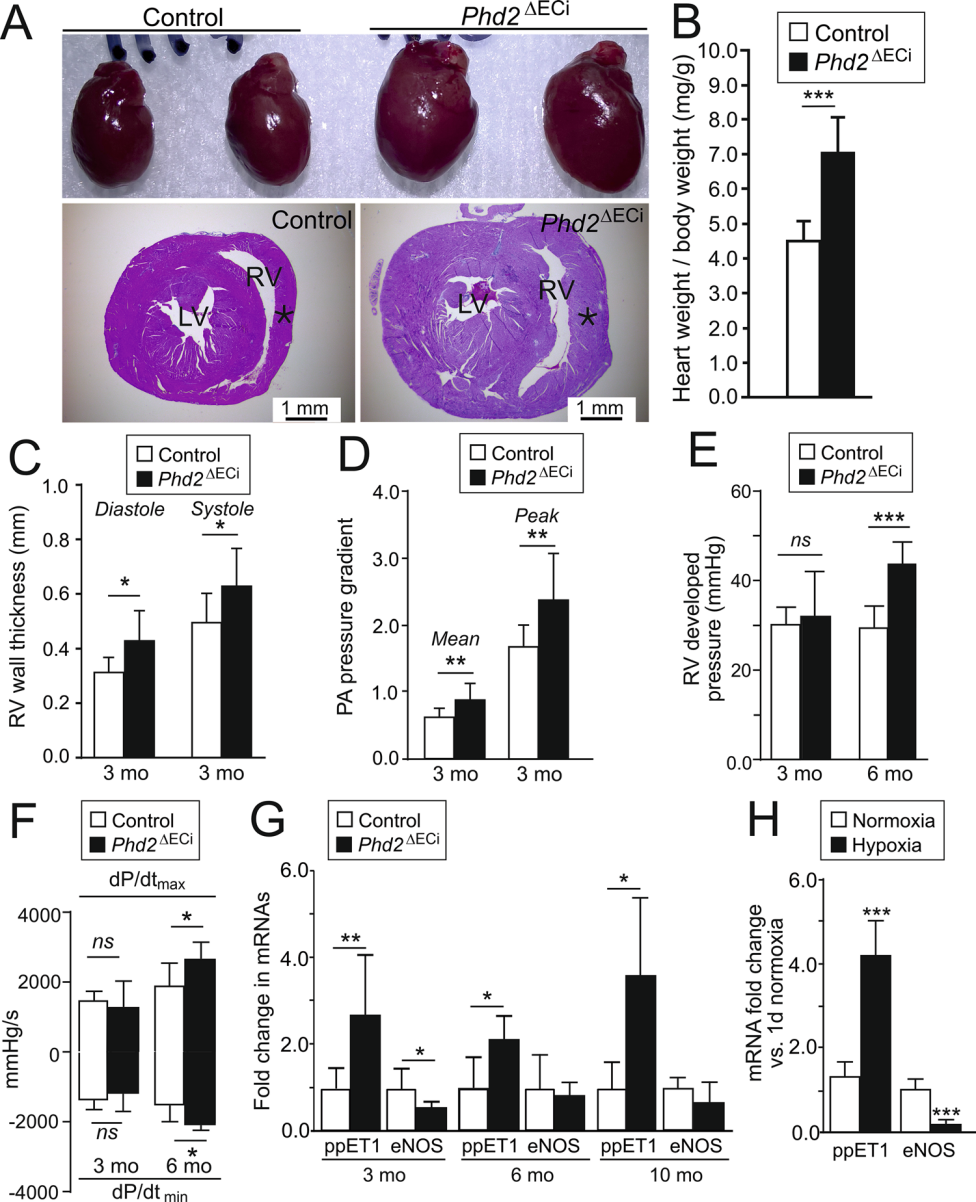
Cardiac hypertrophy, elevated pulmonary arterial and right ventricular pressures in *Phd2*^∆ECi^ mice. **A** Representative images of 10-month-old mice hearts (top) indicating increased cardiac size in *Phd2*^ΔECi^. Masson trichrome staining of transverse histological sections (bottom) show increased thickness of right ventricular (RV) wall (asterisk) without evidence of fibrosis or ventricle dilation. LV, left ventricle. **B** Increased heart weight to body weight ratio in 3 to 10 month-old *Phd2*^ΔECi^ mice, *n* = 8–19 mice/group. **C**, RV wall thickness measured by M-mode in echocardiography at 3 months of age. **D** Pulmonary artery (PA) pressure calculated from Doppler ultrasound data. **E** RV developed pressure. **F** Rate of RV pressure development. **G** qPCR analysis of endothelial PAH marker genes in mouse lungs, *n* = 5–8 mice/group. **H** qPCR analysis of endothelial PAH marker genes in ECs cultured in normoxic or hypoxic conditions for 7 days. C-F, 6–10 mice/group; **H** the experiments were repeated 3 to 4 times. Mean ± SD. *P < 0.05, **P < 0.01 and ***P < 0.001 in *t*-test. Mo, month

### *Phd2*^∆ECi^ mice develop increased pulmonary artery and right ventricular pressure, right ventricular hypertrophy and increased cardiac contractile function

As *Phd2*^∆ECi^ showed no readily apparent phenotype at 3 or 6 months of age, we first investigated possible indications in more detail in the oldest (10-month-old) *Phd2*^∆ECi^ cohort. In contrast to the inducible global deletion of floxed *Phd2 *allele [5] in *Phd2*^∆ECi^ we observed no erythema, hemorrhages, venous congestion, nor premature mortality. Nevertheless, gravimetric analysis revealed cardiac hypertrophy (Fig. 2A, B) as previously found in mice where PHD2 was deleted in ECs starting in early embryonic development [6–8]. As RV hypertrophy is a commonly used diagnostic measure of PAH [26], we next measured RV dimensions in living mice using transthoracic echocardiography. This indicated increased RV wall thickness in *Phd2*^∆ECi^ mice at 3 months of age (Fig. 2C). In PAH RV hypertrophy develops as a compensatory response to chronic pressure overload that was investigated using Doppler velocimetry (indicating flow velocity in the pulmonary artery) [27] and directly from the RV. In Doppler, an indication for increased pulmonary artery pressure was observed at 3 months of age (Fig. 2D). Intraventricular catherization measurements showed that at 3 months age RV pressure was increased in some, but not all *Phd2*^∆ECi^ mice, and there was a significant correlation between the highest and lowest pressure readings and RV wall diameter in systole (Pearson *r* = 0.84, *P* = 0.018, *n* = 7 XY pairs). 6-month-old *Phd2*^∆ECi^ mice demonstrated a significant increase in RV pressure (43.94 ± 4.70 mmHg vs. 29.87 ± 4.39 mmHg in controls, *n* = 6–10 mice/genotype) as well as enhanced inotropic and lusitropic function (Fig. 2E-F), the latter suggesting compensatory rather that pathological cardiac remodeling.

### Upregulation of pulmonary arterial hypertension marker genes in the lungs of* Phd2*^∆ECi^ mice

As increased RV and pulmonary arterial pressure are typical indications of PAH [28] we next measured transcript levels of genes previously implicated in this condition (Fig. 2G). Endothelial nitric oxide synthase (eNOS, a vasodilator via NO), was downregulated in 3-month-old *Phd2*^∆ECi^ lungs, but not in the older age groups, whereas endothelin I (ppET1, a vasoconstricting factor) was upregulated in all three age groups. In ECs cultured in a hypoxic environment (1% O_2_) for 7 days, ppET1 was upregulated and eNOS downregulated indicating an EC intrinsic PHD2-dependent hypoxic response (Fig. 2H).

### Age-related thickening of respiratory membrane and progressive interstitial fibrosis in *Phd2*^∆ECi^ lungs without arterial remodeling

**Fig. 3:**
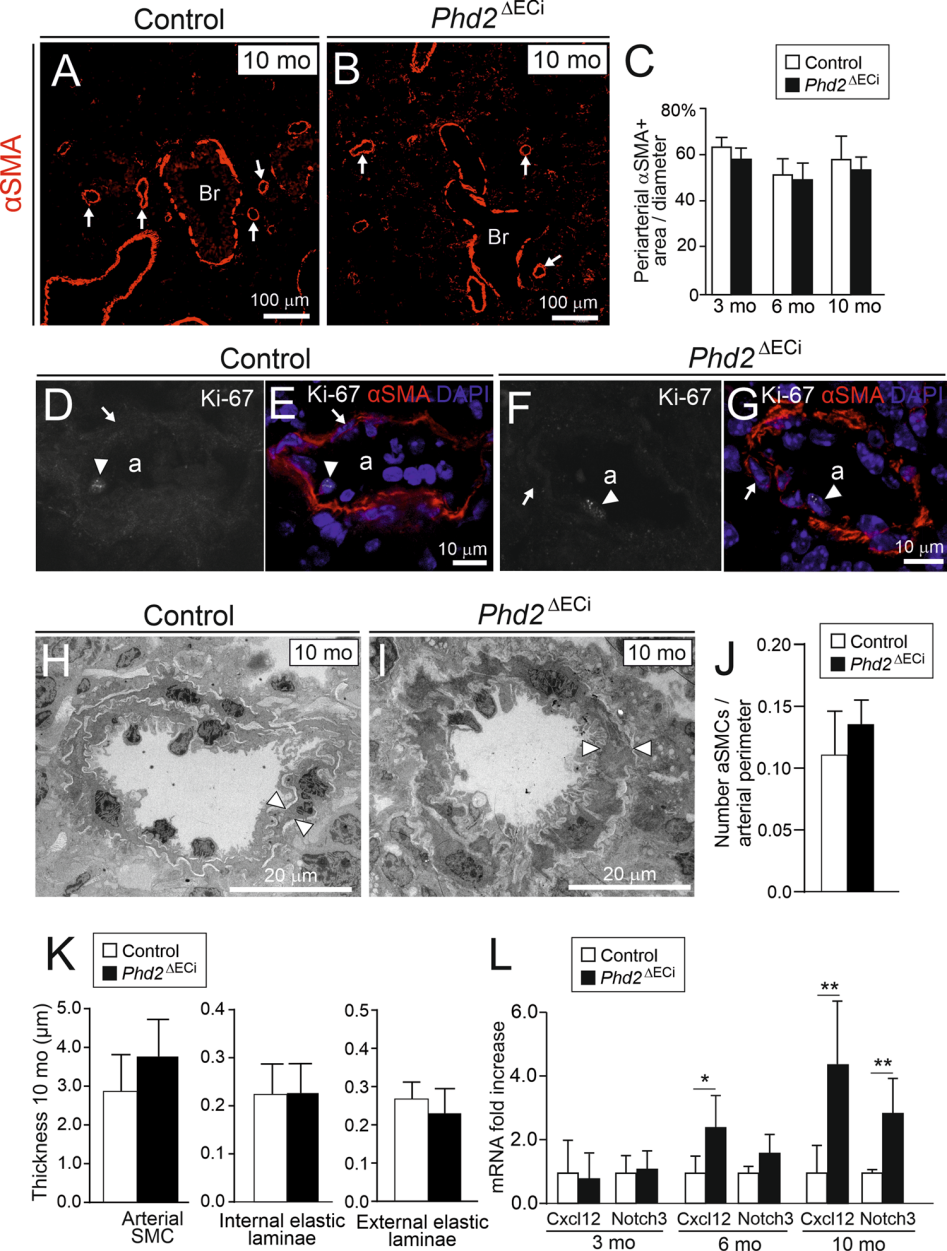
Lack of arterial smooth muscle proliferation, arterial wall and perivascular matrix remodeling in *Phd2*^ΔECi^ pulmonary arteries/arterioles. **A** and** B** Lung sections of 10-month-old mice immunostained with alpha smooth muscle actin (αSMA). Arrows point to αSMA positive arterioles; bronchus (Br). **C** Area of αSMA positive cells around arteries and arterioles, *n* = 5 mice/group, 10 arteries/mouse calculated. **D–G**, Arterioles (a) stained with Ki-67, αSMA and DAPI. EC and aSMCs nuclei are indicated by arrowheads and arrows, respectively. There was no change in proliferation marker Ki-67 between the genotypes. **H** and **I** Representative TEM micrographs of pulmonary arterioles, thickness of single aSMC is indicated by arrowheads. **J** Number of aSMCs/perimeter of arteriole. **K** Thickness of arterial layers in 10-month-old mice. *n* = 5–7 mouse/group (1–4 arteries/mouse). **L** Expression of SMCs signaling molecules Cxcl12 and Notch3 in the lungs. *n* = 5–8 mice/group. Mean ± SD. *P < 0.05, **P < 0.01 in *t*-test. Mo, month

Current dogma is that PAH occurs as a consequence of obliterative pulmonary vascular remodeling involving aSMC proliferation, tunica media thickening and periarterial fibrosis increasing vascular resistance [28]. In addition, arterial remodeling is a common finding in PAH caused by chronic hypoxia [8, 29, 30]. To confirm the presence of arterial remodeling also in *Phd2*^∆ECi^ mice, we carefully investigated the histology and ultrastructure of pulmonary arteries and peripheral arterioles. To our surprise we observed no changes in the number of muscularized arteries or in arterial αSMA staining in *Phd2*^∆ECi^ mice (Fig. 3A–C). Furthermore, based on Ki-67 staining aSMC proliferation rate was similarly low in *Phd2*^∆ECi^ (3 positive nuclei out of 493 aSMCs, *n* = 5 mice) and in control mice (1 positive nucleus out of 385 aSMCs, *n* = 4 mice) (Fig. 3D–G). Ultrastructural analyses of pulmonary arterioles with lumen diameters < 40 μm (measured from narrowest point) revealed no significant changes in medial wall thickness (*P* = 0.140, *n* = 5–7 mice/group) or in the number of aSMCs (*P* = 0.176, *n* = 5–6 mice/group) in 10-month-old mice (Fig. 3H–K) or in internal and external laminae (Fig. 3K), the thickness of which was previously shown to increase in hypoxic conditions [31]. Previous studies using developmental deletion of *Phd2* in ECs (E7.5 onwards) proposed a model where increased Cxcl12 [6] and Notch3 [25] promote pulmonary aSMC proliferation. After 10-month deletion both pathways were upregulated in *Phd2*^∆ECi^ lungs (Fig. 3L), indicating that the same SMC stimulating pathways were activated, however, increased expression of these genes was not sufficient for aSMC proliferation in *Phd2*^∆ECi^ mice.

**Fig. 4:**
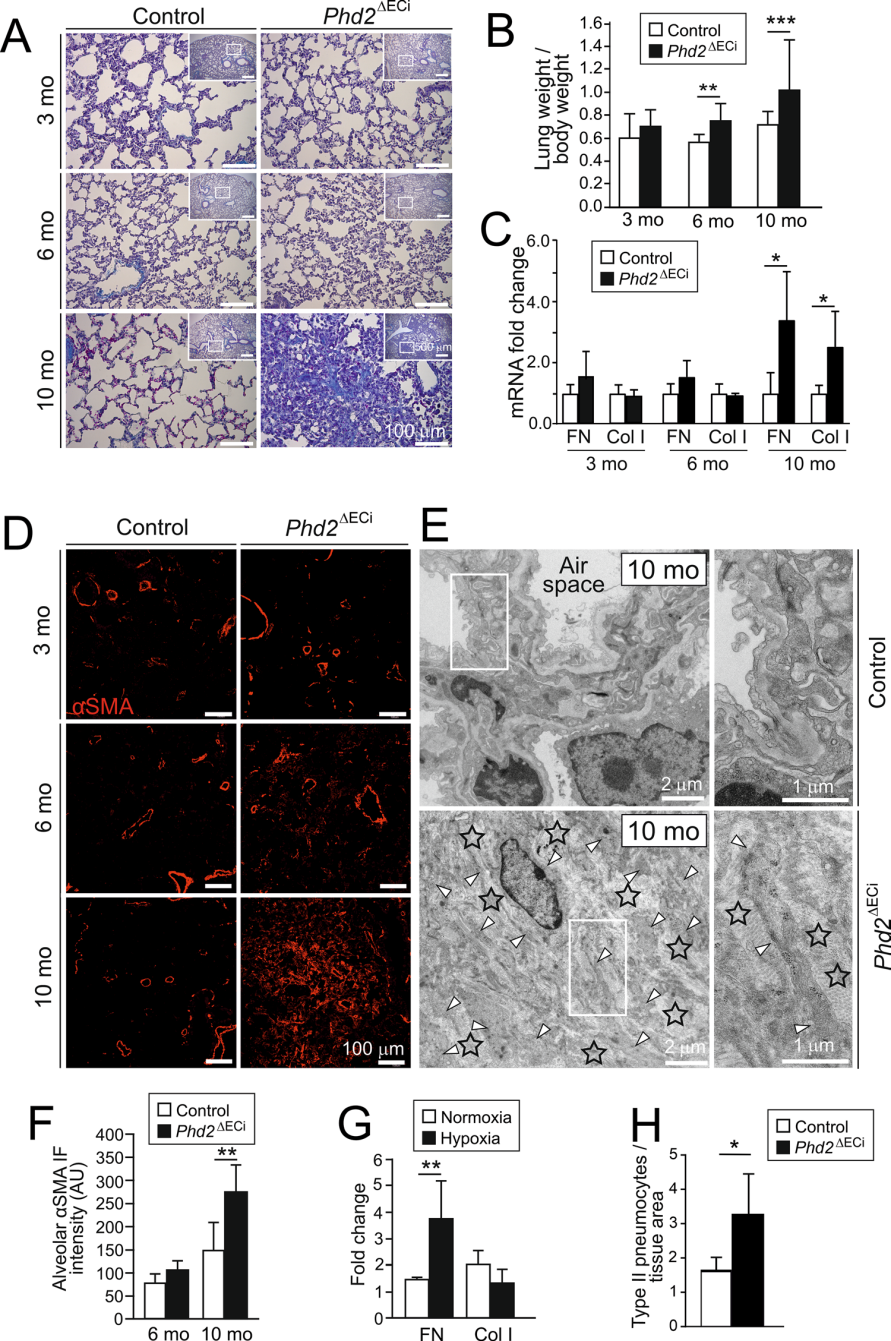
Lung fibrosis in aged *Phd2*^ΔECi^ mice. **A** Masson trichrome staining of lung sections show fibrotic foci (blue) in 10-month-old *Phd2*^∆ECi^ mice. Lower magnification images in the insets indicate area of detail analysis. **B** The lung weight to body weight ratio (%) ((lung weight/body weight) × 100), *n* = 8–19 mice/group. **C** qPCR analysis of lung fibrosis marker genes fibronectin (FN) and type I collagen (Col I), *n* = 5–8 mice/group. **D** Increased number of αSMA positive cells in 10-month-old *Phd2*^ΔECi^ lung tissue. Note that αSMA positive cells are evenly distributed and not preferentially located near arteries (image data quantified in **F**). **E** Ultrastructure of fibrotic area in 10-month-old *Phd2*^∆ECi^ shows loss of alveolic structure, increased fibrillar collagen matrix (asterisks) and numerous myofibroblastic cells (arrow heads) as identified based on myofilament rich cytoplasm, presence of dense plaques and lack of BM. **F** quantification of non-arterial αSMA staining in 10-month-oldmice lungs, *n* = 4–5 mice/group. **G** qPCR of fibrosis marker genes in ECs cultured in hypoxia for 7 days in comparison to normoxia, *n* = 3 experiments. **H** Quantification of type II pneumocytes in *Phd2*^ΔECi^ lungs as identified in TEM sections based on surfactant granules, cell size and location (*n* = 5–6 mice). Mean ± SD. *P < 0.05, **P < 0.01, *** *P* < 0.001 in *t*-test. Mo, month

**Fig. 5:**
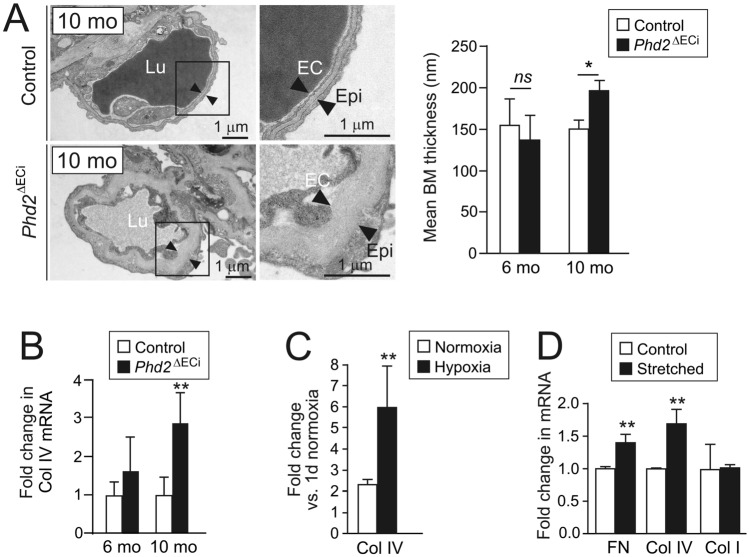
*Phd2* deletion and hypertension mimicking mechanical stretching results in capillary basement membrane thickening. **A** Respiratory membrane is formed by alveolar epithelial (Epi) and capillary ECs that are separated by alveolar capillary BM (black arrowheads) which main constituent is Col IV. Note thickening of BM in 10-month-old *Phd2*^∆ECi^ mice. Image data quantified on the right, *n* = 5–8 mice/group. **B** qPCR of Col IV in the lungs, *n* = 5–8 mice/group. **C** qPCR analysis of Col IV mRNA in ECs cultured in hypoxia for 7 days and compared to normoxia, *n* = 3 experiments. **D** Expression of Col IV, fibronectin (FN), and type I collagen (Col I) mRNAs in vitro model for hypertension induced EC stretching. ECs were exposed to stretching for 24 h and results were normalized to non-stretched controls, *n* = 3 experiments. Mean ± SD. *P < 0.05, **P < 0.01 in *t*-test. Mo, month

Masson trichrome staining, pulmonary weight, and the key transcripts relevant to pulmonary fibrosis (fibronectin, FN; collagen I, Col I) were increased after 10 month *Phd2* deletion time indicating progressive pulmonary pathology (Fig. 4A–C). In 10-month-old *Phd2*^∆ECi^ several fibrotic foci were evident showing interstitial αSMA immunofluorescence, clusters of activated fibroblasts/myofibroblasts and increased fibrillar collagen deposition in the alveolar septum but not around arteries (Fig. 4D–F). To investigate direct involvement of ECs in the fibrotic response due to hypoxia, ECs were exposed to a low oxygen atmosphere that increased FN but not Col I mRNA expression (Fig. 4G). As an additional indication of lung injuries in 10-month-old *Phd2*^∆ECi^ mice, we found an increased number of type II pneumocytes [32] (3.29 ± 1.15 vs. 1.66 ± 0.34 in control, *P* = 0.016 in Welch’s *t*-test, *n* = 5–6 mice/group) (Fig. 4H) and thickened capillary BMs (Fig. 5A). The latter paralleled the upregulation of Col IV mRNA expression (Fig. 5B), the major component of vascular BMs that was also induced in ECs exposed to low oxygen (Fig. 5C). Increased mechanical stress in alveolar capillaries stemming from elevated blood pressure has been implicated in BM remodeling in PH [33]. To isolate the impact of mechanical stress from  PHD2 dependent hypoxic insult, ECs were subjected to pulsatile mechanical stretching mimicking hypertension-induced hemodynamic load. Interestingly, this resulted in upregulation of Col IV and FN in normoxia (Fig. 5D). Col I was not increased in ECs in hypoxia or in stretching. This suggested EC to stromal cell signaling in the fibrotic lungs to stimulate fibrillar collagen synthesis in cell types other than ECs that typically produce BM constituents.

### Right ventricular systolic pressure increases in aged ***Phd2***^∆aSMC^ mice independently from endothelial vascular tone marker genes

**Fig. 6:**
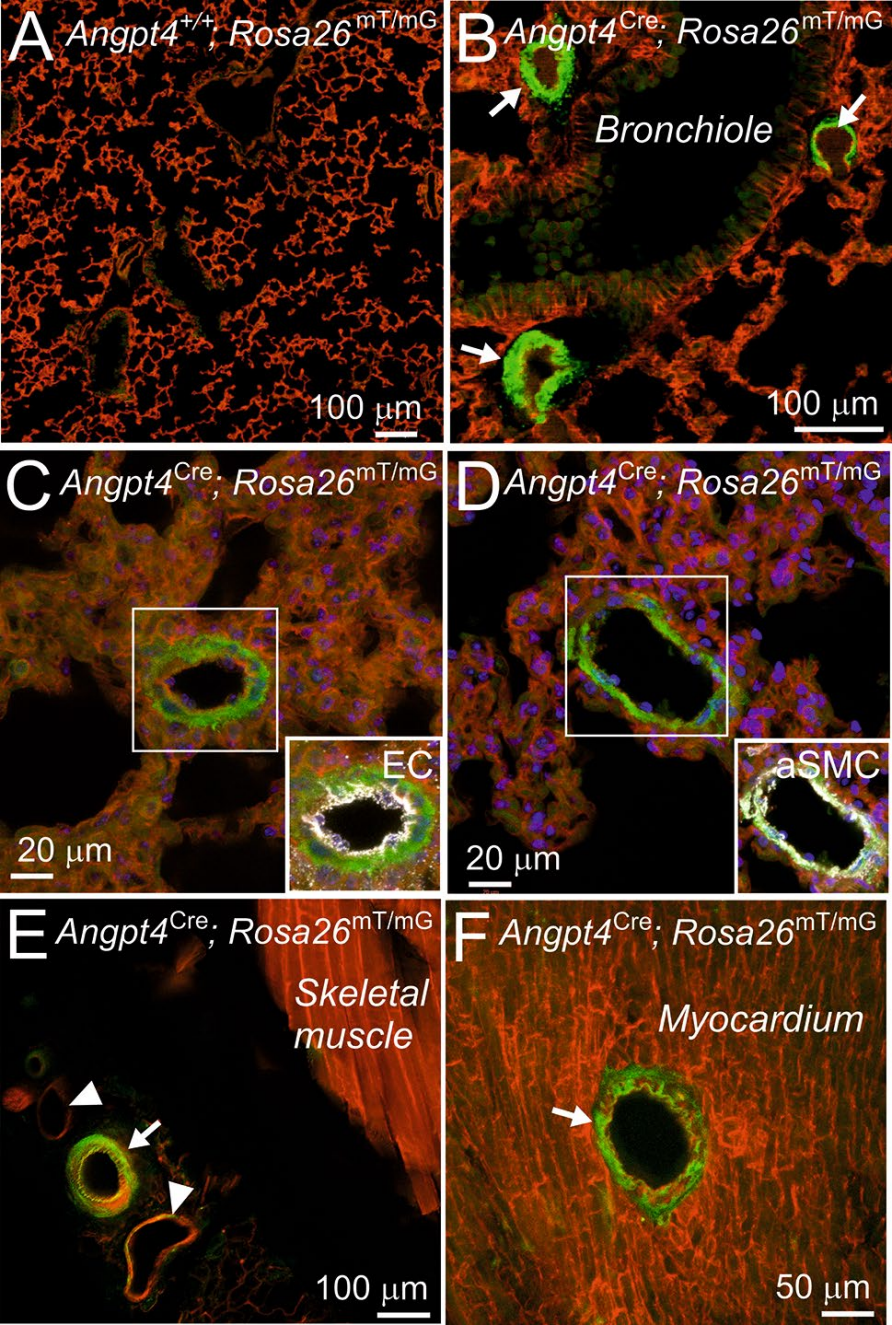
Arterial smooth muscle cell expression of *Angpt4*^Cre^. *Angpt4*^Cre^ mice were crossed with *Rosa26*^mT/mG^ cell lineage tracing line that express cell membrane targeted mTomato prior *Angpt4*^Cre^-induced mGFP (green) expression. **A** *Angpt4*^+*/*+^*; Rosa26*^mTmG^ lung samples as negative control showed ubiquitous mTomato and faint background in the bronchiolar epithelium (green). **B** In *Angpt4*^Cre^*; Rosa26*^mTmG^ lung samples GFP is expressed in arterial smooth muscle cells (arrows). **C** Arterial ECs (pseudo-colored in white) are negative for *Angpt4*^Cre^-induced GFP expression. **D** aSMA staining (white) colocalized with *Angpt4*^*Cre*^-induced GFP in aSMC in arterial media layer. **E** Femoral aSMCs (arrow) are *Angpt4*^Cre^ expressive, veins (arrowheads) are negative or show only some occasional *Angpt4*^Cre^ expressing cells. Skeletal muscle is *Angpt4*^Cre^ negative. **F** Arterial media layer (arrow) is positive for *Angpt4*^Cre^ in the heart, whereas surrounding cardiac muscle is *Angpt4*^Cre^ negative. Samples were collected from 6-month-old mice

The data above suggested that ECs may function as hypoxia sensing cells and that the development of arterial remodeling and lung fibrosis involves other cell types than only ECs. To study the role of  PHD2 in aSMCs in vivo we utilized a novel *Angpt4*^Cre^ knock-in allele. In our previous work we found *Angpt4* expression in aSMCs of the small intestine mesentery in adult mice [34]. To characterize the spatiotemporal expression pattern of Angpt4 in more detail, *Angpt4* mRNA expression was investigated by qPCR and its cellular source analyzed in the fate mapping *Angpt4*^Cre^*; Rosa26*^mT/mG^ mice. In qPCR analysis, the earliest *Angpt4* expression in the lungs was detected at P9. In *Angpt4*^Cre^*; Rosa26*^mT/mG^ mice *Angpt4*^Cre^ expressing cells were observed at P11 around arteries, but not at the earlier time points analyzed (P1 and P4). In *Angpt4*^Cre^*; Rosa26*^mT/mG^ mice, GFP expression was located in the aSMCs in the medium-sized distributing pulmonary arteries and in arterioles ranging in diameter from approximately 20 μm to 100 μm (Fig. 6A–D). In addition, GFP was detected in some sparse, fibroblast-like cells (Supplemental Fig. 3). Importantly, and in contrast to commonly used SMC Cre diver lines [15], *Angpt4*^Cre^ was not expressed in visceral or bronchial SMCs, and Cre expression did not leak into skeletal or cardiac muscle cells (Fig. 6E, F). This data identified *Angpt4*^Cre^ as a potential Cre driver for pulmonary aSMCs and was consistent with the Tabula Muris single cell transcriptome data base, indicating *Angpt4* expression in the generic “stromal cell” population in the lungs at three months of age, but not in pulmonary ECs or epithelial cells [35].

**Fig. 7:**
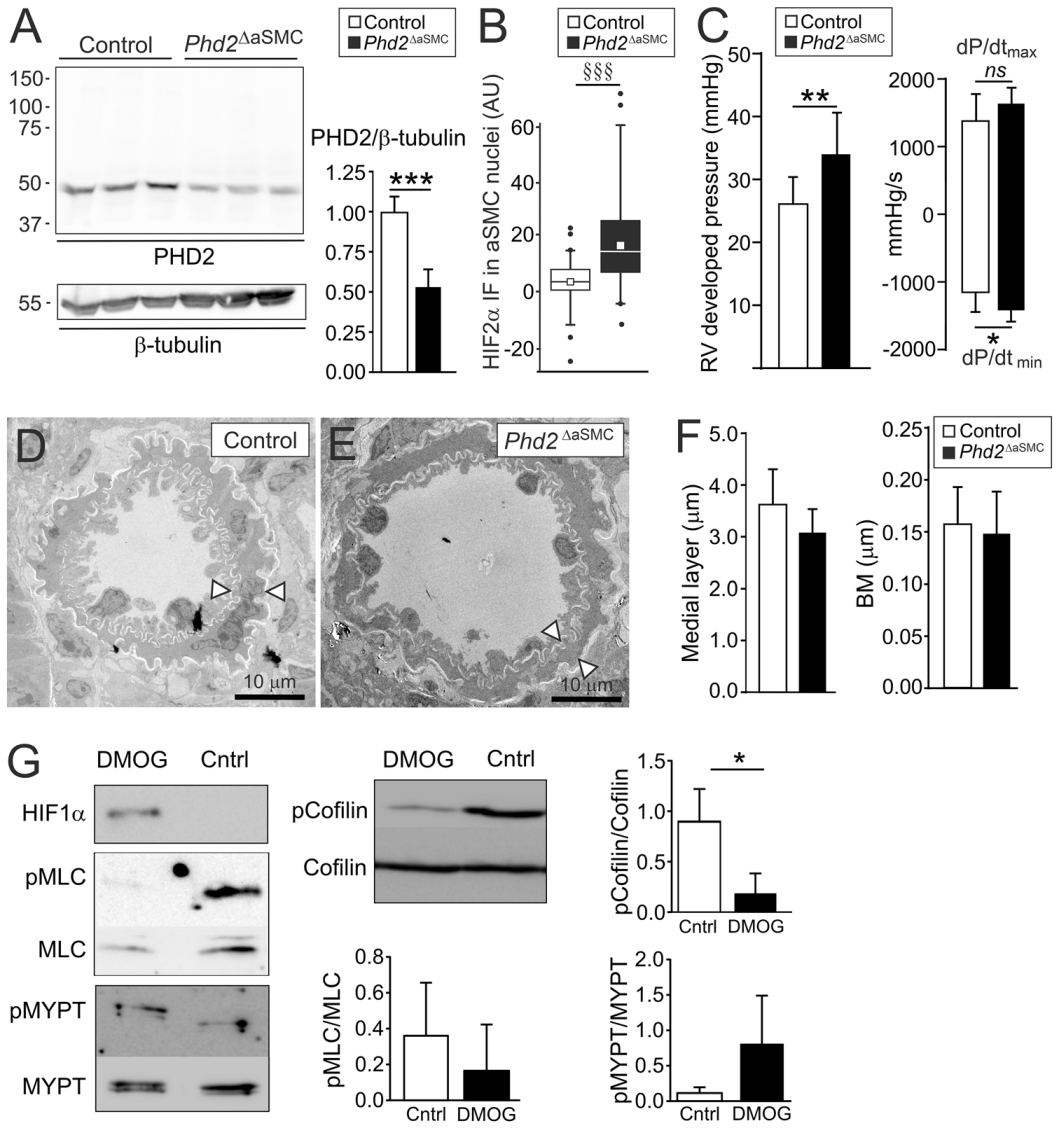
*Phd2* deletion from arterial smooth muscle cells caused modest pulmonary hypertension in 8-month-old mice without arterial remodeling. **A** Western blot analysis (scale in kDa) of lung lysates showed reduced amount of PHD2 protein in *Phd2*^ΔaSMC^ mice. Residual PHD2 is expected from Cre-negative cells. Signal intensities quantified on the right, control set to 1, *n* = 6 mice/genotype. **B**, Relative HIF2α immunofluorescence intensity in aSMC nuclei, *n* = 67 and 58 aSMC nuclei from three control and three *Phd2*^ΔaSMC^ mice. Median (line), average (square), 75th quartile (box), 5th and 95th percentile (whiskers), outliers (•). **C**, RV developed pressure and rate of RV pressure development. **D–F** No differences in the thicknesses of the medial layer of pulmonary arteries (*n* = 4–5 mice/genotype) or alveolar capillary BM (*n* = 3–6 mice/genotype) between control and *Phd2*^ΔaSMC^ mice. **G** Western blot analysis of phosphorylation levels of proteins indicated from cultured aSMCs treated with PHD inhibitor DMOG or DMSO (control), *n* = 3 independent experiments. Mean ± SD. ***P < 0.001, **P < 0.01, *P < 0.05 in *t*-test. ^§§§^*P* < 0.001 in Mann–Whitney test

*Phd2*^loxP^ was crossed with the *Angpt4*^Cre^ allele to generate *Angpt4*^Cre/+^*; Phd2*^loxP/loxP^ mice (thereafter *Phd2*^∆aSMC^). Analysis of lung samples from 8-month-old *Phd2*^∆aSMC^ mice indicated reduced PHD2 protein level and increased HIF2α immunofluorescence intensity in aSMC nucleus ensuring *Phd2* deletion (Fig. 7A-B, Supplemental Fig. 2). Interestingly, *Phd2*^∆aSMC^ mice showed increased RV pressure (Fig. 7C) and relaxation time, but no pulmonary arterial remodeling or other histopathological changes (Fig. 7D–F). Furthermore, and in contrast to *Phd2*^∆ECi^, *Phd2*^∆aSMC^ showed no significant change in endothelial vascular tone marker genes ppET1 and eNOS or SMC stimulating factors Cxcl12 [6] and Notch3 [25] (data not shown).

### PHD inhibition in cultured arterial smooth muscle cells results in altered actin dynamics

Increased RV pressure in *Phd2*^∆aSMC^ suggested increased aSMC contractility that is primarily regulated by myosin light chain (MLC) phosphorylation and actin polymerization [36]. To investigate the underlying mechanism, we inhibited PHDs using dimethyloxalylglycine (DMOG) [37] in aSMCs. DMOG stabilized HIF but did not increase MLC or MYPT phosphorylation (Fig. 7G). In addition to pMLC driven crossbridge cycling, actin filament assembly, stimulated by non-phosphorylated cofilin enhances SMC contraction [21, 38]. Interestingly, in the DMOG-treated aSMCs the relative level of non-phosphorylated cofilin was clearly increased (Fig. 7G).

## Discussion

**Fig. 8:**
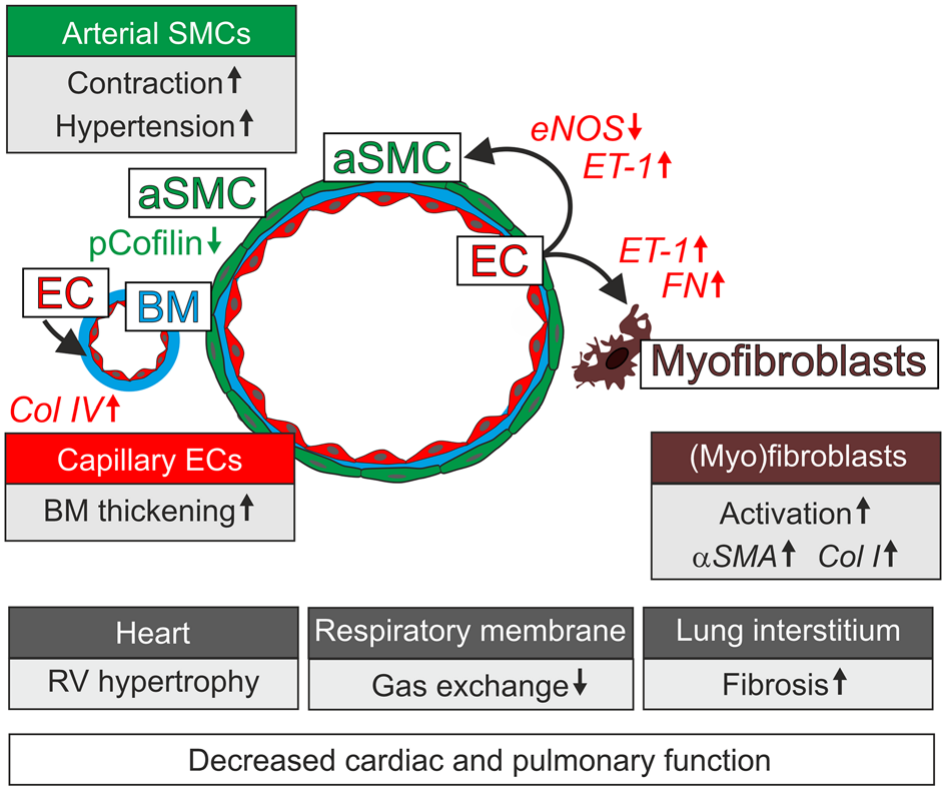
PHD2-dependent mechanisms in pulmonary hypertension and fibrosis. In ECs PHD2 deficiency results in HIF stabilization and dysregulated expression of vascular tone regulators (eNOS, ET-1) causing hypercontraction of aSMCs and pulmonary hypertension. RV hypertrophy develops secondary to the chronic pressure overload. PHD2 deletion resulted in increased expression of  Col IV in ECs and thickened respiratory membrane. In contrast to ECs, in aSMCs PHD2 deletion elevated RV pressure via activated cofilin/actin polymerization without changes in vascular tone regulators. EC produced ET-1 may also promote myofibroblast activation and fibrosis

PAH is one form of pulmonary hypertension characterized by sustained pulmonary arterial vasoconstriction and pathological arterial wall remodeling. The primary cause remains elusive in most cases and there is no curative treatment. In the lungs hypoxia results in arterial vasoconstriction to adjust ventilation to perfusion [39]. The PHD2-regulated HIF hypoxia signaling system has been identified as a potent vasoconstrictive pathway and when chronically activated it results in PAH in genetically modified mice [6–8]. Although still incompletely understood, the outcome of PHD2 inhibition/HIF stabilization likely depends on the timing, extent, and cell type(s) when and where it occurs. This is exemplified in mice carrying a hypomorphic *Phd2* allele resulting in lowered *Phd2* expression in various cell types (in the lung tissue 35% to 45% of WT) and stabilization of HIF1α and HIF2α, but no alterations in the lung architecture or development of fibrosis [40]. In-depth understanding of the cellular mechanism and signaling pathway by which PHD2 deficiency can result in PAH may help to find molecular targets to alleviate the symptoms in pulmonary diseases. Furthermore, identification of the mechanisms underlying the detrimental effects of PHD2 inhibition is also important from a translational point of view, as PHD inhibitors are in clinical development and the first of them has been recently accepted for the treatment of anemia [20]. Due to shortcomings in previous genetic approaches (the inability to distinguish developmental defects from the pathogenesis occurring in the matured lungs and the lack of desirable cell-type specificity for genetic modifications in ECs and aSMCs), the cellular mechanisms that determine how PHD2 deficiency initiates and promotes pulmonary artery hypertension remains incompletely known. This is particularly true in the case of aSMCs, the key cell type regulating pulmonary vascular tone via vessel dilation/constriction, periarterial matrix production and arterial wall remodeling. In our study, we take advantage of a novel *Angpt4*^Cre^ allele that to the best of our knowledge, is the first Cre driver for the generation genetic modifications in the aSMCs. In addition, using inducible, EC-specific gene deletion postnatally (*Phd2*^∆ECi^) by applying the *Chd5*^CreERT2^ line [22, 23], we were able to separate pathological changes affecting adult vascular homeostasis from the developmental phenotypes occurring in the embryos. Figure 8 summarizes the identified dysregulated signaling pathways and cellular responses in vascular and interstitial cells and their potential contribution to the decreased cardiac and pulmonary function in PAH.

Analyses of the *Phd2*^∆ECi^ mice revealed deletion time-dependent, progressive changes in the lungs and heart including signs of PAH (increased pulmonary artery and RV pressure, adaptive RV hypertrophy, expression of PAH associated genes) and indicators of increased alveolar wall stress and injury (type II pneumocyte hyperplasia, focal fibrosis, increase in lung weight) [32, 41, 42]. Nevertheless, *Phd2*^∆ECi^ did not show arterial wall thickening, aSMC proliferation or apparent occlusion of pulmonary arteries, that are common histopathological findings in PAH. The lack of obliterative arterial remodeling in *Phd2*^∆ECi^ model (Cre expression induced at one month of age) was different from non-inducible *Tie2-Cre* [6] and *VE-Cadherin-Cre* models [6–8] in which Cre expression started at early development (at E7.5). We propose that the differences in phenotypes reflect differential roles of PHD2 in embryonic and in adult blood vasculature [1, 2, 4] and that the extent and time course of the activation of hypoxia signaling also contribute to the severity of vascular disease. This suggestion is in line with the exposure of mice to hypoxia at different ages developing more severe phenotypes when exposed at birth than as mature animals [5, 43, 44].

It is widely accepted that aSMCs have a key role in hypertension, however, due to lack of a specific aSMC Cre-deleter line it has not been possible to investigate the in vivo importance of aSMCs intrinsic responses. Furthermore, at the molecular level, how the activation of the hypoxia signaling pathway results in increased SMCs contractility and proliferation has been controversial. Suggestive for a SMC intrinsic hypoxia response is the observation that HIF1α protein is increased in cultured pulmonary aSMCs isolated from idiopathic PAH patients [45]. On the other hand, deletion of the floxed *Phd2* allele using the smMHC/Myh11 promoter driven Cre [13] enhanced PAH in hypoxia but not in normoxia, thus suggesting a secondary rather than an initiatory role for aSMCs in PAH pathogenesis. As a potential shortcoming, the smMHC/Myh11 promoter is active in all vascular (arterial, venous) and visceral SMCs and also in lung pericytes [15] that likely influence the phenotype. In contrast to Chen *et al.* [13] *Phd2*^∆aSMC^ showed mild hypercontractility (increase in RV pressure) in normoxia but was not associated with lung pathologies as observed in *Phd2*^∆ECi^. This indicated that PHD2 deletion in aSMC can increase contractility to elevate RV pressure, but PHD2 deletion in aSMC alone is not sufficient to result in obliterative arterial remodeling, lung damage or fibrosis. Interestingly, in vitro analysis of HIF stabilization in aSMCs suggested that increased contractility may occur via actin polymerization-related tension development via activated cofilin and independently from MLC phosphorylation. The results are in line with a previous report of HIF1α dependent dephosphorylation of MLC in pulmonary aSMCs [46]. Moreover, there is consistent evidence that actin polymerization plays an essential role in SMC contraction and tension development [47]. One of the main regulators in this context is cofilin, which binds to filamentous actin providing more free barbed ends for actin polymerization [48] and tension development [21]. In aSMCs PHD2 inhibition increased active (dephosphorylated) cofilin thus suggesting that PHD2 deficiency may potentiate vascular pathologies by increasing the contractility of aSMCs and structurally “sensitize” the aSMC cytoskeleton for ET-1 (produced by ECs) induced vasoconstriction [38].

Pulmonary interstitial fibrosis in aged *Phd2*^∆ECi^ mice was an additional pathological manifestation of postnatal deletion of *Phd2*. BM remodeling and fibrotic changes could develop as a response to increased hemodynamic stress [49, 50] and EC to stromal cell signaling. In the latter mechanism upregulated ET-1 plays an important role as it induces hypertension, myofibroblast differentiation and fibrosis [42, 51]. Among the known vasoconstrictor agents ET-1 was consistently upregulated in *Phd2*^∆ECi^ mice from the earliest age group analyzed and also in ECs after one week of culture in low oxygen environment, which indicates an early role for ET-1 in pulmonary pathologies due to chronic activation of PHD2/HIF signaling.

Studies aimed to investigate the pathophysiology of PAH have thus far mainly focused on large vessels while the potential involvement of pulmonary microvasculature has been overlooked. Our ultrastructural analysis of *Phd2*^∆ECi^ mice revealed thickening of the respiratory membrane, a two-cell layer formed by alveolar epithelial cells and capillary ECs separated by Col IV, the expression of which was increased in *Phd2*^∆ECi^ mice and in ECs cultured in low oxygen. The rate of gas exchange through this specialized BM layer is inversely proportional to its thickness [52] and the observed alterations likely exacerbate respiratory symptoms. This assumption is in line with clinical studies revealing reduced pulmonary membrane diffusion capacity in the patients with elevated pulmonary arterial pressure indicating functional impairment of the alveolocapillary membrane [53]. Hypertension imposes hemodynamic stress on the alveolar wall, which has been shown to associate with remodeling of the BM [50, 54]. In ECs grown in a normoxic atmosphere, we also found upregulation of Col IV following 20% pulsatile stretching mimicking pathological hypertension [55, 56]. This suggests that respiratory membrane thickening is likely a net result of EC hypoxia sensing and hypertension induced wall stress in alveoli. This causality can be deduced from the law of LaPlace, which states the inverse relationship of wall stress to wall thickness and has been evidenced in clinical and experimental models [33, 54, 57].

In addition to the animal models discussed above, chronic HIF activation due to a loss-of-function mutation in PHD2 [58], a hypomorphic mutation in HIF-binding von Hippel-Lindau protein (leading to HIF stabilization) [59, 60] and a gain of function mutation in HIF2α [61] are associated with cardiopulmonary pathologies. Furthermore, ECs in the plexiform lesions of PAH patients showed increased HIF levels in situ and in vitro [62, 63]. Identification of the role of PHD2 in the matured vasculature in *Phd2*^∆ECi^ mice is not only necessary for better understanding of pathogenesis of pulmonary vasculopathies, but also important to predict possible long-term side effects of PHD2 inhibitors that have recently entered the clinics for anemia treatment [20, 64]. In this respect it is interesting to note that in contrast to severe PAH phenotypes, as one might expect based on previous results [6–8], pulmonary and cardiac complications typical for PAH have been reported only in one patient thus far [65]. While our *Phd2*^∆ECi^ model did not recapitulate all PAH pathologies, we did observe progressive vascular resistance and lung damage in aged mice. Based on our results, long-term clinical follow-up would be important to fully evaluate the safety of prolyl hydroxylase inhibitors.

## Material and methods

Generation of the *Phd2*^loxP^ allele is described in Rosendahl *et al*., (Rosendahl A-H, Raasakka M, Laitala A, Railo A, Miinalainen I, Heljasvaara R, Mäki JM, Myllyharju J, manuscript in preparation). *Chd5*^CreERT2^ mice were kindly provided by Ralf Adams, Max Planck Institute for Molecular Biomedicine, Department of Tissue Morphogenesis, Muenster, Germany and are described previously [22]. *Phd2*^loxP^ and *Chd5*^CreERT2^ mouse lines were crossed to generate the *Chd5*^CreERT2^; *Phd2* ^loxP/loxP^ line. *Chd5*^CreERT2^; *Phd2*^loxP/loxP^ mice were administered with four to five injections of tamoxifen (4–5 mg, T5648, Sigma-Aldrich) intraperitoneally or oral gavage starting from one month of age to generate *Phd2*^∆ECi^*.* Litter mates *Chd5*^CreERT2^; *Phd2*^+/+^, *Chd5*^CreERT2^; *Phd2*^+/-^, or *Chd5*^CreERT2^; *Phd2*^loxP/+^ received the same tamoxifen administrations and were controls for *Phd2*^∆ECi^ mice. No alteration was observed in mice having at least one PHD2 wild type allele (*Phd2*^+^) as also previously noted [66]. RVs were analyzed by echocardiography and the mice euthanized at the age of 3, 6 and 10 months to collect tissue samples. To confirm the efficiency and cell type specificity of the Cre/loxP recombination *Chd5*^CreERT2^; *Rosa26*^mTmG^ Cre-reporter mice [67] were treated with the same tamoxifen dosage as above and GFP expression was investigated by confocal microscopy as an indication for Cre/loxP mediated recombination from tissues of 10-month-old mice. To achieve aSMCs specific *Phd2* deletion in the lungs, a floxed *Phd2* allele was crossed with an *Angpt4*^Cre^ mouse line [34] to generate *Phd2*^ΔaSMC^ (*Angpt4*^Cre/+^*; Phd2*^loxP/loxP^). To confirm efficiency and cell type specificity of the Cre/loxP recombination *Angpt4*^Cre^ were crossed with *Rosa26*^mTmG^ mice. *Angpt4*^Cre^*; Phd2*^loxP/loxP^ RVs were analyzed by echocardiography and the mice euthanized at the age of 8 months to collect tissue samples as described below. In gravimetric analysis tissue weight was normalized with the body weight and presented using the formula; tissue weight/bodyweight×100.

### Histological analysis

Tissue samples were fixed overnight in 10% formalin or 4% paraformaldehyde. Fixed tissues were embedded in paraffin, processed and stained with hematoxylin and eosin and Masson's trichrome by routine methods. Myofibroblasts and SMCs were visualized using Cy3-αSMA (C6198, Sigma-Aldrich) antibody, ECs with endomucin (sc-65495, Santa Cruz Biotechnology). Ki-67 was used as an immunohistochemical marker for cell proliferation (TEC-3, M7249, Dako). Fluorescently labelled sections were imaged using confocal microscopy: Olympus FluoView 1000 UPLSAPO 20×/0.75 or UPLSAPO 60×/1.35 oil objectives or with Leica SP8 Falcon by using HC PL APO 63×/1.40 OIL CS2 objective or with Zeiss LSM 780 by using Plan-Apochromat 20×/0.8 or i Plan-Apochromat 63×/1.4 oil objectives. Nuclear HIF2α was quantified from frozen lung sections stained with HIF2α (#109616, Abcam) antibody. In this assay, arteries were identified from the tissue sections based on their morphology and αSMA staining. Arterial SMCs and EC nuclei were manually segmented based on DAPI staining and their mean fluorescence intensity was measured using Fiji processing package of Image J2 software. To correct for technical variations in staining intensities between the slides the mean fluorescence intensity of non-relevant nuclei was subtracted from the nucleus of interest. Histological sections were imaged using Leica DM LB with C Plan 4×/0.1 objective and Leica DCF320 camera or Zeiss Axio Imager.M2m by using EC Plan-Neofluar 40×/0.75 objective and Zeiss Axiocam 506 color camera. αSMA positive cells were quantified by analyzing relative intensity of αSMA. In cell culture, HIF2α nuclear localization was quantified by measuring mean immunofluorescent intensity (#109616, Abcam). To correct for technical variations in staining intensities between the samples, mean intensity measured from cytoplasm was subtracted from the mean intensity measured from the nucleus. Intensity analyses were done using Fiji.

### Western blot analysis

Lung tissue samples from mice were homogenized in RIPA lysis buffer supplemented with protease inhibitor cocktail (P8340, Sigma-Aldrich). Western blot analysis was performed using anti-PHD2 (NB100-2219 Novus Biologicals and #4835S Cell Signaling), HIF1α (NB100-479 Novus Biologicals) and HIF2α (NB100-122 Novus Biologicals) antibodies. Anti-β-actin (A2066 Sigma-Aldrich) and anti-β-tubulin antibodies (Sigma T4026) were used as a loading control. Phospho-MYPT1 (Thr696, 5163T), MYPT1 (2634T), phospho-myosin light chain 2 (Thr18/Ser19, 3674T), myosin light chain 2 (D18E2, 8505), phospho-Cofilin (Ser3, 77G2) and Cofilin (D3F9, XP® 5175T) antibodies were all from Cell Signaling Technology.

### Transmission electron microscopy (TEM)

Specimens were fixed in 1% glutaraldehyde and 4% formaldehyde in 0.1 M phosphate buffer, pH 7.4, then postfixed in 1% osmium tetroxide, dehydrated in acetone and embedded in Epon LX112 (Ladd Research Industries). 150 nm sections were stained with toluidine blue to select regions of interest. 80 nm sections were cut with a Leica Ultracut UCT microtome and imaged using Tecnai Spirit transmission electron microscope (Fei Europe) and Quemesa CCD camera (Olympus Soft Imaging Solutions GMBH).

### Morphometrical analysis of pulmonary vasculature

Arterial wall thickness measurements were performed from TEM micrographs at a × 480 magnification. Medial layer thickness was measured from eight locations to cover the whole circumference of an artery. Internal and external elastic laminae measurements were taken from 13 to 21 locations per artery. Number of SMCs/perimeter of arteries was counted by measuring perimeter based on internal elastic lamina. Capillary BM thickness was determined from at least 20 images (magnification × 2900) captured randomly throughout the lung tissue section. The measurements were taken from four locations covering the whole circumference of a capillary. All measurements were averaged to present a single value per artery or capillary. Type II pneumocytes were counted from 3908 μm^2^ TEM micrographs (magnification × 690) based on their morphological characteristics and normalized to total tissue area by using thresholding-based segmentation in Fiji.

### Quantitative RT-PCR

Total RNA was extracted using an RNeasy Mini or Fibrous Tissue Mini kit (Qiagen) following the manufacturer´s protocols. For cDNA synthesis, 1 to 3 μg of RNA was mixed with random hexamers and oligo-dT, 200 U M-MLV Reverse Transcriptase (Promega), 20 U RiboLock RNase inhibitor (Thermo Scientific), 0.5 mM dNTP and reaction buffer (50 mM Tris–HCl, 75 mM KCl, 3 mM MgCl_2_), incubated at +42 °C for 1 h, then at +70 °C for 15 min and diluted 1:3 to 1:10 in sterile H_2_O and used in qPCR (2 μl/reaction). Real-time qPCR was performed using a Stratagene mx3005P qPCR instrument (Agilent Technologies) and Brilliant Ultra-Fast SYBR QPCR Master mix (Agilent Technologies). DNA primer sequences are indicated in the Supplemental Table I.

### Right ventricular pressure measurements and echocardiography

A Vevo 2100 (Fujifilm-Visualsonics) was used to acquire echocardiographic parameters with MS-550D transducer (40 MHz, 40 μm axial, 90 μm lateral resolution) under isoflurane anesthesia (4% for induction and 1,5% for maintenance). B-mode, M-mode and Doppler imaging were used to obtain RV size and function as well as aortic and pulmonary flow. All analyses were performed by an experienced sonographer who was blinded for the experimental groups. For open chest RV pressure measurements, the animals were anesthetized with ketamine and xylazine (112.5 mg/kg and 15 mg/kg, respectively) and intubated. The chest was cut open with a high temperature cauterizer (Fine Science Tools). A pressure–volume sensor 1.2F (Transonic Science) was introduced into the RV from the apical side. The pressure was recorded and analyzed with Labscribe V2 (iWork Systems inc.). Pressure values were extracted after 5 min of baseline recording.

### Cell culturing

Human ECs (PromoCell, C0155C) were cultured in Human Endothelial Cell Growth Medium supplemented with growth supplement (Cell Applications), 1% penicillin/streptomycin (PS) (Sigma-Aldrich) and 10% FBS (HyClone). Before seeding the cells, the culture plates were coated with Attachment Factor (Cell Applications) for at least 30 min in 37 °C. For hypoxia experiments, ECs were cultured in 1% oxygen for 7 days in a hypoxia workstation InVivo400 (Ruskinn). Mouse ECs (C57-6023, Cell Biologics) were cultured in M1168 (Cell Biologics) supplemented with M1168-Kit. Mouse embryonic fibroblasts NIH3T3 (ATCC) were cultured in DMEM-GlutaMAX™-I (Gibco) with 1% PS and 10% FBS. Human aSMCs (Cell Applications) were cultured in Smooth Muscle Cell Growth Medium (SMCGM, Cell Applications). To promote maturation to a contractile phenotype the SMCGM was changed to Human SMC Differentiation Medium (Cell Applications) for 5 days. To inhibit the activity of PHD2, the cells were treated with 1 mM or 3 mM of dimethyloxalylglycine, N-(methoxyoxoacetyl)-glycine methyl ester (DMOG, Calbiochem) or 25 µM Roxadustat (FG-4592, Cayman Chemicals) for 24 h. Equivalent volume of DMSO was used as a control.

### Cell stretching experiments

Human ECs (PromoCell, C0155C) were cultured until confluence in Col I coated BioFlex plates (Flexcell inc.) and washed two times with culture medium devoid of serum and growth supplement. The cells were exposed to 20% equibiaxial stretch in 1 Hz cycles for 24 h in DMEM supplemented with 0.5% FBS.

### Statistics

All data is presented as mean ± standard deviation. Normal distribution and equal variances were tested using the Shapiro–Wilk and *F*-test, respectively. The two-tailed unpaired *t*-test (normally distributed data) and Mann–Whitney test were used for comparison of two groups. In the case of inequal variances Welch’s correction was used. Statistical tests were done using Origin Pro software. The test used is indicated in the Figure legends.

## Supplementary Information

Below is the link to the electronic supplementary material.

Supplementary file1 (PDF 8346 kb)
