# Supplementary material for: PHD2 deletion in endothelial or arterial smooth muscle cells reveals vascular cell type-specific responses in pulmonary hypertension and fibrosis
Source: Angiogenesis. 2022 Jan 8;25(2):259–74. doi: 10.1007/s10456-021-09828-z (PMC9054891; doi:10.1007/s10456-021-09828-z)
Supplement: Supplementary file 1 — Supplementary file1 (PDF 8346 kb) [file 10456_2021_9828_MOESM1_ESM.pdf]

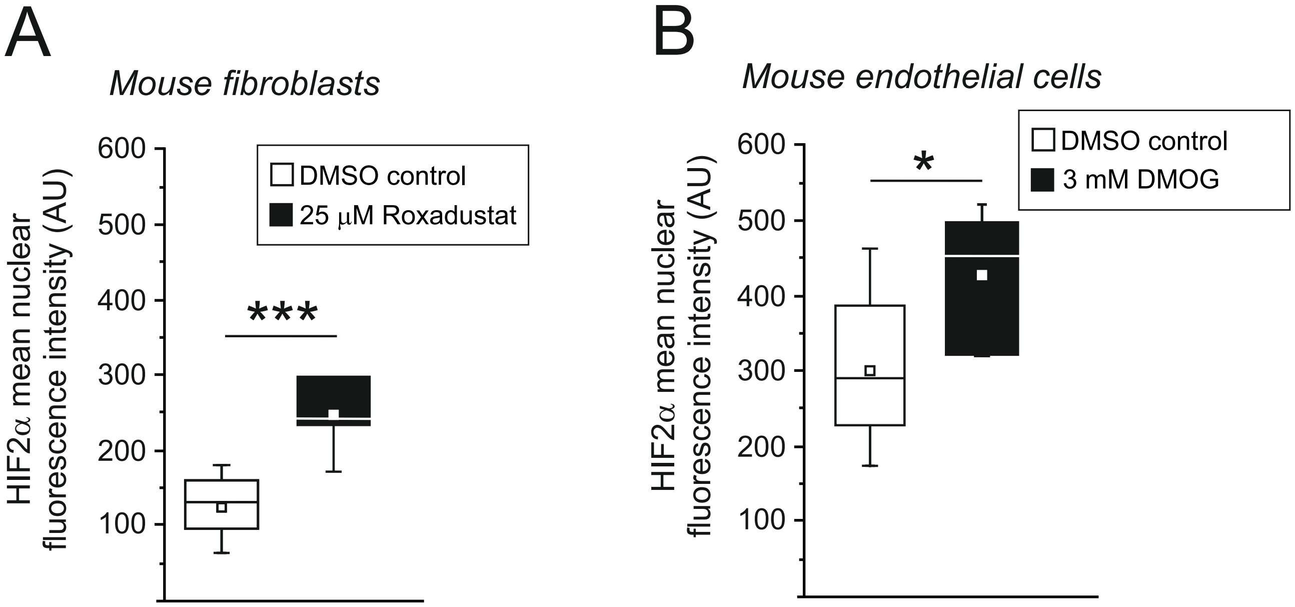

Supplemental Figure 1. Validation of HIF2α antibody (#109616, Abcam) for quantitative analysis of nuclear translocation of HIF in mouse cells following the inhibition of PHDs. HIF2α mean immunofluorescence intensity in A) mouse NIH3T3 fibroblast and B) mouse primary brain microvascular endothelial cells. n = 6 to 16 replicates, each represent the average of three randomly selected 40x microscopy fields, one field containing 10 to 40 cells. Mean nuclear immunofluorescence intensity was normalized to cytosolic staining and quantified by Fiji. Median (line), average (square), 75th quartile (box), 5th and 95th percentile (whiskers), ***P<0.001, *P<0.05 inhibitor vs. control in t-test.

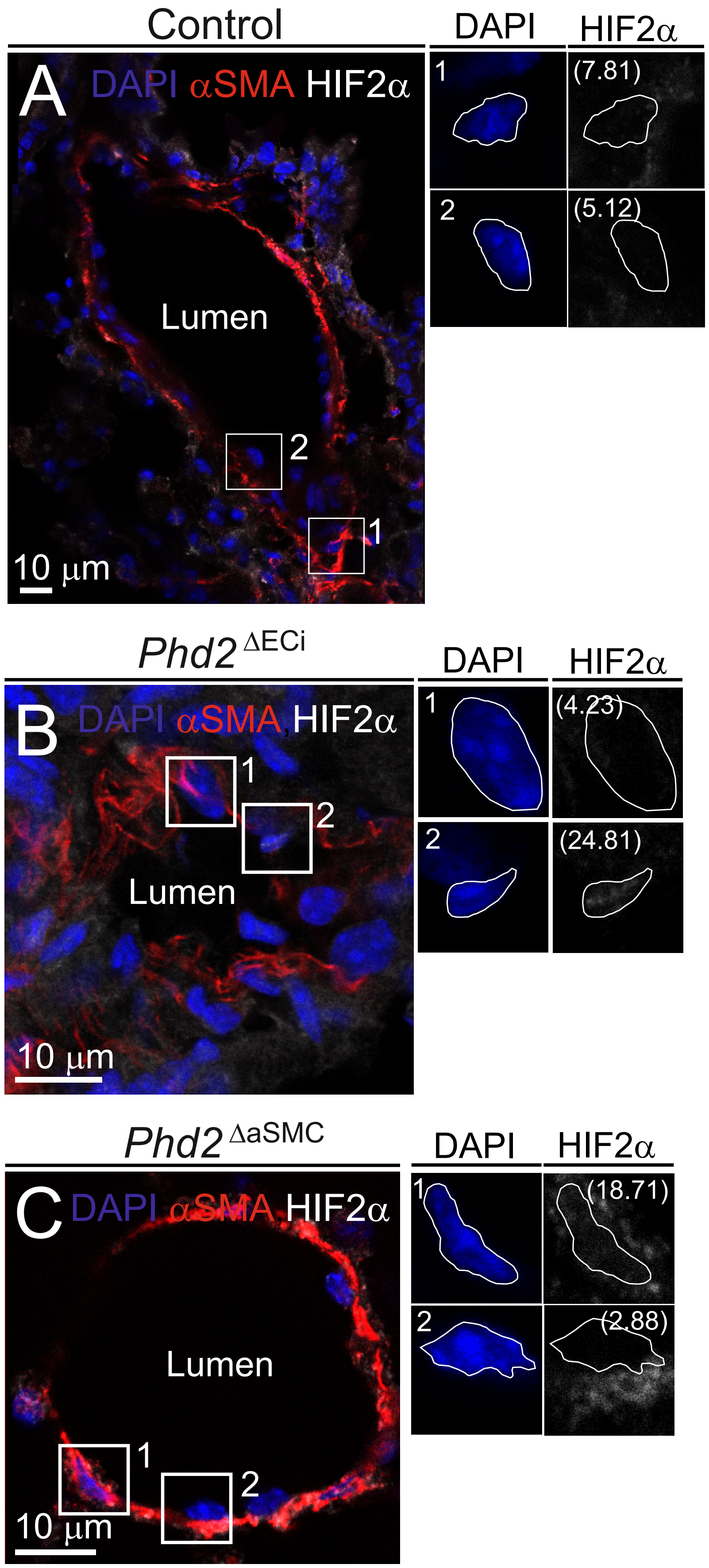

Supplemental Figure 2. A-C, pulmonary arteries stained with DAPI (nuclear counterstaining) and antibodies against αSMA (for aSMCs) and HIF2α (for stabilization and nuclear translocation of HIF) in arterial SMCs (1) and ECs (2). HIF2α mean immunofluorescence intensity indicated in the inserts (parenthesis). Image data quantified in Figures 1D and 7B.

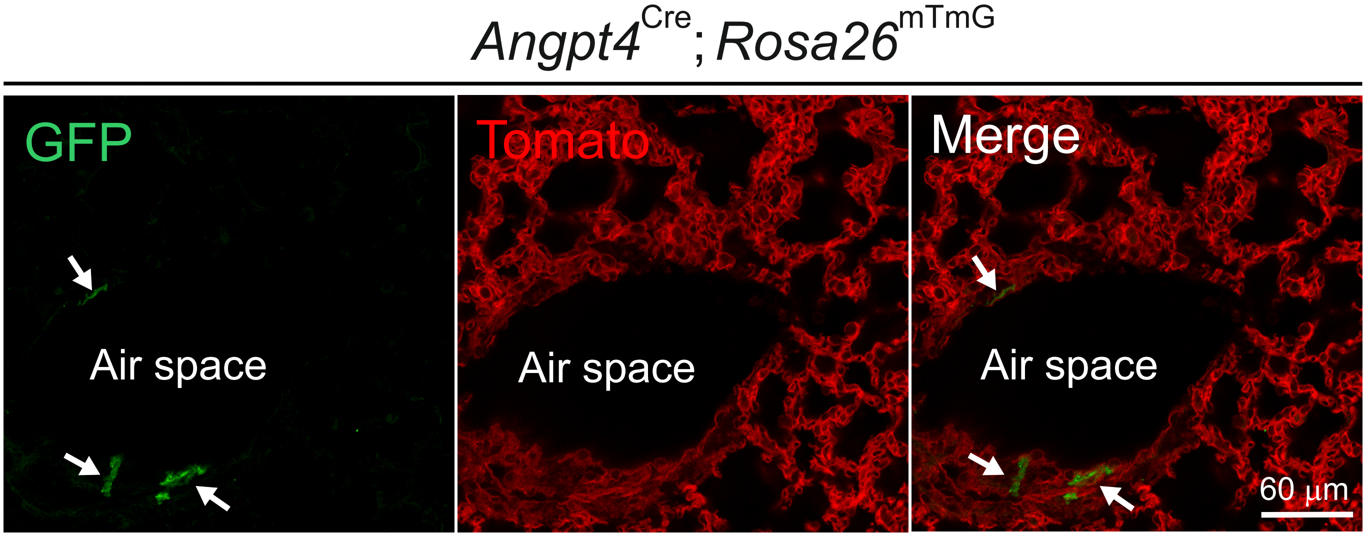

Supplemental Figure 3. *Angpt4*^{Cre} mice were crossed with *Rosa26*$^{mT/mG}$ cell lineage tracing line expressing mTomato prior Cre -induced mGFP expression. In addition to aSMCs, *Angpt4*^{Cre} is expressed in same sparse, fibroblast-like cells (arrows) in the lung parenchyma. Sample collected from 10-month-old mouse.

Supplemental Table I. Primer sequences for RT-PCR

**Primer**	**Source**	**Sequence**
ppET1 Forw	Mouse	ACTTCTGCCACCTGGACATC
ppET1 Rev	Mouse	GTCTTTCAAGGAACGCTTGG
eNOS Forw	Mouse	TGAAGATCTCTGCCTCACTCATG
eNOS Rev	Mouse	AGTCTCAGAGCCATACCAGAATGGTT
Col4a1 Forw	Mouse	CTGGCACAAAAGGGACGAG
Col4a1 Rev	Mouse	ACGTGGCCGAGAATTTCACC
Col1a1 Forw	Mouse	TGACTGGAAGAGCGGAGAGT
Col1a1 Rev	Mouse	GTTCGGGCTGATGTACCAGT
FN Forw	Mouse	ATCTGGACCCCTCCTGATAGT
FN Rev	Mouse	GCCCAGTGATTTCAGCAAAGG
β-actin Forw	Mouse	TGTTACCAACTGGGACGACA
β-actin Rev	Mouse	GGGGTGTTGAAGGTCTCAAA
CXCL12 Forw	Mouse	CCAAGAGTACCTGGAGAAAGC
CXCL12 Rev	Mouse	AGTTACAAAGCGCCAGAGCA
Notch3 Forw	Mouse	TGAACAACGTGGAGGCTACC
Notch3 Rev	Mouse	GCAGCCTGTCCAAGTGATCT
Phd2 Forw	Mouse	GGCAAAGCCCAGTTTGCTGACATTG
Phd2 Rev	Mouse	TGAGTTCAACCCTCACACCTTTCTCAC
COL1A1 Forw	Human	GGTTTCGACTTCAGCTTCCTG
COL1A1 Rev	Human	TCACCAGTCTCCATGTTGCAG
COL4A1 Forw	Human	GTGCAAGGCAATGAACGGGC
COL4A1 Rev	Human	GCCTCACACACAGCACACCT
eNOS Forw	Human	GCACAG GAAATGTTCACCTAC
eNOS Rev	Human	CACGATGGTGAC TTTGGCTAG
FN Forw	Human	CCTCGAAGAGCAAGAGGCAG
FN Rev	Human	GCTTCAGGTTTACTCTCGCA
GAPDH Forw	Human	ACAGTCAGCCGCATCTTCTT
GAPDH Rev	Human	ACGACCAAATCCGTTGACTC
PHD2 Forw	Human	GCACGACACCGGGAAGTT
PHD2 Rev	Human	CCAGCTTCCCGTTACAGT
ppET1 Forw	Human	TATCAGCAGTTAGTGAGAGG
ppET1 Rev	Human	CGAAGGTCTGTCACCAATGTGC
